# Protein changes in abalone foot muscle from three geographical populations of *Haliotis diversicolor* based on proteomic approach

**DOI:** 10.1002/ece3.2128

**Published:** 2016-04-27

**Authors:** Guilan Di, Xiulian Miao, Caihuan Ke, Xianghui Kong, Hui Li, Weiwei You

**Affiliations:** ^1^ College of Fisheries Henan Normal University Xinxiang 453007 China; ^2^ State Key Laboratory of Marine Environmental Science College of Ocean and Earth Sciences Xiamen University Xiamen China; ^3^ College of Life Sciences Liaocheng University Liaocheng 252059 China

**Keywords:** geographical population, *Haliotis diversicolor*, muscle, proteomic, two‐dimensional gel electrophoresis

## Abstract

Using two‐dimensional gel electrophoresis, the foot muscle proteome of three geographical populations of *Haliotis diversicolor* were examined, with a total of 922 ± 21 protein spots detected in the Japanese population (JJ), 904 ± 25.6 in the Taiwanese population (TT), and 936 ± 16.2 in the Vietnamese population (VV). Of these, 254 spots showed differential expression and 85 protein spots percentage volumes varied more than twofold. Both “genotype” and “spot” analysis of variance approaches significantly showed differences among the three populations. Hierarchical clustering analysis showed that TT and VV clustered together followed by clustering with JJ, which is consistent with their geographical location. Following matrix‐assisted laser desorption/ionization time‐of‐flight mass spectrometry, 30 differentially expressed proteins involved in major biological processes including energy production and storage and stress response were identified. Of these proteins, proteins pertaining to muscle contraction and muscle protein regulation showed highest expression levels in VV samples. Proteins involved in energy production and storage, including ATP synthase beta subunit, fructose‐1,6‐bisphosphate aldolase, arginine kinase, enolase, triosephosphate isomerase, and tauropine dehydrogenase, showed diverse expression patterns among the three populations. For stress‐responsive proteins, the expression of heat shock protein 70 was JJ > VV > TT. The expression pattern of Cu/Zn‐superoxide dismutase was JJ > VV > TT. Overall, these results may aid in the detection of new differentially expressed proteins within three different abalone populations.

## Introduction

Abalones are marine gastropods distributed worldwide along coastal waters in tropical, subtropical and temperate areas (Klinbunga et al. 2009; Amano et al. 2010). The Chinese abalone aquaculture production accounted for more than 80% total production all over the world in 2014, and the small abalone *Haliotis diversicolor* is one of commercially important abalone species in China (FAO 2014) cultured in Asia. The phenotypic traits for three geographically isolated populations of small abalone, Japan (JJ), Taiwan (TT), and Vietnam (VV), were tested from early juvenile on Day 5 to adults on Day 420 (You et al. 2009). At the grow‐out stage, shell lengths of the Japanese and Taiwanese populations were 7.48% and 15.72% larger than that of the Vietnamese population at Day 420. For the entire rearing period, the Japanese population displayed the highest survival (78.3 ± 5.34%), being significantly higher than the Taiwanese (12.6 ± 4.13%) and Vietnamese (15.7 ± 4.62%) populations (You et al. 2009). However, the molecular mechanisms for the differences in growth and survival rates in these three groups are still vague.

The study of genetic variability among individuals, populations and species and the evolutionary forces driving these variations is becoming an active area of research (Diz and Skibinski 2007). In recent years, modern genetic analyses have attempted to identify relationships between molecular genetic markers and physiological phenomena (Nie et al. 2014). While proteins are effectors of genetic information and have specific biological functions, alterations at the genetic level are not necessarily visible at the protein level (Xiang et al. 2013). Proteins determine phenotypic traits, with the phenotype being the final form of gene expression, and in most cases, this is not directly linked to the gene locus. Proteins can be thought of as snapshots of genomic expression (Jin et al. 2014), with individually expressed proteins treated as quantitative inheritance characters, as is most often the case with nonmodel species (Vasemägi and Primmer 2005). While several methods can be applied to study the genetic characteristic leading to phenotypic differences at the molecular level (Xie et al. 2006), the selection of only one or a few proteins as markers can inadequately reflect the complexity of the genetic information.

Examination of the proteome, also referred to as the molecular phenotype, has been insightful in population, phylogenetic, and evolutionary studies (Feder and Mitchell‐Olds 2003; Navas and Albar 2004; Biron et al. 2006). Some proteomic approaches are able to quantitatively multiplex thousands of proteins for comparison between samples (Jones et al. 2013). This approach enables the identification of differentially expressed proteins between two or more samples, thus providing insight into cellular mechanisms (Anderson and Anderson 1998).

Of the multiplexing approaches, two‐dimensional gel electrophoresis (2‐DE) has been used to determine protein expression between two mussel populations, living under two different ecological conditions (Diz and Rolán‐Alvarez 2014). This approach has also been used to compare global protein expression between two related species of marine mussels, *Mytilus edulis* and *M. galloprovincialis*, growing in different geographical habitats. For the 420 compared protein spots, 15 and 22 proteins were identified as up‐regulated proteins in *M*. *edulis* from the Netherlands and Iberian *M. galloprovincialis*, respectively (López et al. 2002). Furthermore, 2‐DE in conjunction with mass spectrometry methods was utilized to identify whole mantle proteins from patterned and nonpatterned *Meretrix meretrix*, from Yueqing, Zhejiang, East China (Su et al. 2009). Proteomic approaches have been widely used for specie identification, genetic variability examination (López 2005), and many biological systems (López 2007). However, proteomics is usually a neglected level despite the evidence supporting the importance of protein expression patterns study (García et al. 2013). These approaches have been scarcely applied to the examination of marine gastropods recently.

In the present study, a proteomic approach was applied to compare *H. diversicolor* foot tissue protein expression among three geographical populations (Japanese, Taiwanese, and Vietnamese). The aim of this study was to further elucidate foot tissue protein differences among the three geographical populations. Overall, these results will contribute to understanding of the molecular differentiation and future ecological, systematics studies of the three geographical populations.

## Materials and Methods

### Chemicals

IPG, dry‐strips, and pH 4–7 were purchased from Amersham Biosciences (Piscataway, NJ). Chemicals used for electrophoresis were obtained from Bio‐Rad (Hercules, CA) and other analytical grade chemicals were obtained from Sigma (St. Louis, MO).

### Population background and conditioning

Three geographically isolated *H. diversicolor* populations were used in this study. Small abalones were collected from one wild and two hatchery populations (our team research papers You et al. 2011). The Japanese wild population (JJ) was collected from Izu Island, Tokyo. The Taiwanese cultured population (TT), which was originally introduced from Taiwan to mainland China in 1992, and parents of these cultured samples have been propagated in hatcheries over several generations in Fujian Province. The Vietnamese cultured population (VV), which was firstly introduced from Hai phong into China in 2005 and then offspring from the F_2_ generation were used in the experiment (You et al. 2005, 2011).

For each of the three abalone populations (TT, JJ, and VV), 200 abalones were strictly isolated in the farming process and maintained in the Dongshan Haitian Hatchery, Fujian province under the same culturing conditions. Abalones were fed *Gracilaria* sp. and the water temperature was kept at 26–28°C. The abalone sizes were not significantly different. For each population (TT, JJ or VV), muscle proteins were extracted from 18 abalones (10‐month‐old individuals), with every six abalone samples pooled to generate three replicates to ensure reproducibility.

### Abalone muscle protein extraction

The foot muscle was advocated as previously described (López 2005; Diz and Skibinski 2007; Di et al. 2013), with 20 mg muscle sample from each abalone crushed in liquid nitrogen with a pestle and mortar. The powder was suspended immediately in 1 mL Trizol and sample preparations were performed as previously described (Di et al. 2013). The dry pellet was resuspended in isoelectric focusing (IEF) redissolved buffer (7 mol/L urea, 2 mol/L thiourea, 4% [w/v] CHAPS, 40 mmol/L Tris). Protein concentrations were measured according to the method of Protein 2‐D Quant kit (GE Healthcare, Piscataway, NJ, USA). The 18 samples obtained from each group were divided into three subgroups, each containing six abalone muscles to yield 120 *μ*g proteins per subgroup, and stored at −70°C.

### Two‐dimensional gel electrophoresis

The obtained precipitate was solubilized in buffer containing 7 mol/L urea, 2 mol/L thiourea, 4% (w/v) CHAPS, 1% (w/v) DTT, 0.5% (v/v) ampholyte (pH 4–7), and 35 mmol/L Trisbase. Approximately, 120 *μ*g (analytical run) or 1.0 mg (preparative gel for mass spectrometric analysis) of total protein was used for each run. The first dimension of 2‐DE, IEF, was carried out on 18 cm IPG strips with a linear gradient (pH 4–7; Amersham Pharmacia Biotech, Piscataway, NJ, USA) in a horizontal electrophoresis apparatus (Bio‐Rad).

IPG dry‐strips were rehydrated directly with rehydration buffer (8 mol/L urea, 2% (w/v) CHAPS, 20 mmol/L DTT, 0.5% (v/v) IPG buffer (pH 4–7), and 0.01% (w/v) bromophenol blue) at 50 V. Isoelectric focusing was then started at 100 V for 2 h, 200 V for 2 h, 500 V for 1 h, 1000 V for 2 h, 4000 V for 2 h, and finally to 8000 V until the voltage reached 50,000 V. Before completing the second dimension, the IPG strips were gently soaked twice for 16 min in equilibration solution I [6 mol/L urea, 50 mmol/L Tris–HCl buffer (pH 8.8), 2% SDS, 30% glycerol, and 1% w/v dithiothreitol] followed by 16 min in equilibration buffer II (same as buffer I, but dithiothreitol replaced with 2.5% iodoacetamide). The second dimension of gel electrophoresis was carried out on 12.5% polyacrylamide gels (20 cm × 20 cm × 1.5 mm) using a protean Xi Cell (Bio‐Rad). Equilibrated strips were placed onto gels to perform the SDS‐PAGE at 16°C. The separation was carried out at 12.5 mA/gel for 30 min and then 25 mA/gel for about 5.5 h until the dye front reached the bottom of the gel. For analytical gels, the protein spots were visualized with silver nitrate, while preparative gels were stained with Coomassie Brilliant Blue G‐250 (Bio‐Rad, Hercules, CA, USA). All 2‐DE gels from all populations were compared simultaneously.

### Image acquisition and analysis

The 2‐D gels were scanned to generate TIFF files using an Image Scanner (UTA‐1100; Amersham Biosciences), with spot intensity differences analyzed using the PDQuest 8.0 software package (Bio‐Rad). The detected spots were matched between gels and the parameters were adjusted to ensure that only true spots were identified. The background was subtracted and the filtered images were edited to correct possible errors and remove any inaccurate spots. Spot intensity levels were normalized by expressing the intensity of each protein spot as a proportion of the total protein intensity in a gel (relative volume, % vol). Three gels were obtained and matched for each abalone population, with only well‐resolved spots used and those in overlapping areas, streaked areas or near the edges being discarded.

### Statistical analysis

Statistical data analysis for population comparisons were conducted using SPSS version 13.0 software (SPSS Inc., Chicago, IL,USA). As previous described (Karp et al. 2005), only spots presented in all three technical replicates were analyzed (Diz and Skibinski 2007). To compare the three populations, a two‐sided analysis of variance (ANOVA) was used, to include “spot” and “genotype” factors (Di et al. 2013). Populations were compared by using a least significant difference procedure at the 5% level (*P* < 0.05).

Hierarchical clustering and genetic distances of the three abalone groups were also analyzed. Only well‐resolved spots presented in all technical and sample replicates were included in analysis. Skewing in the spot volume was removed by log_10_ transformation of the spot volume, as described previously (Karp and Lilley 2005). Spots in streaked areas, overlapping, around gel edges, or spots with extremely high/low intensities were discarded by PDQuest software. Protein spots unique for an abalone group were also discarded. A total of 508 protein spot locations were examined and standardized values by PDQuest software (ver. 8.0; Bio‐Rad,Hercules, CA, USA). A dendrogram was constructed from the 508 spots volumes values in three abalone populations using hierarchical clustering R statistical software. Hierarchical clustering was determined with a TMEV heatmap showing spots present in all technical replicates across the gels as previously described (Saeed et al. 2006). Genetic distances between populations were calculated from each protein fraction, allowing two distinct genetic groups of three populations to be distinguished.

Spot intensity analysis was performed using a Student's *t*‐test with two‐side. Three abalone populations were compared in pairs. Differentially expressed proteins were identified as having a spot intensity difference of at least 1.5‐fold among the three abalone populations. For qualitative analysis, spots intensities with at a least a 10‐fold change were considered present/absent.

### Protein identification by mass spectrometry

Protein identification was carried out as previously described (Di et al. 2013, 2015). Briefly, spots of interest were excised and trypsin digested trypsin, with 0.5 *μ*L of extracted sample analyzed by matrix‐assisted laser desorption/ionization time‐of‐flight/time‐of‐flight (MALDI‐TOF/TOF) with a 5800 Proteomics Analyzer (Applied Biosystems, CA, USA). Gel extracts were pooled, dried down and resuspended in a gel matrix using 5 *μ*L 50% (v/v) ACN, and 0.1% (v/v) trifluoroacetic acid (TFA). Next, 0.8 *μ*L of sample was mixed with 0.3 *μ*L of matrix solution (2 *μ*g/*μ*L R‐cyano‐4‐hydroxycinnamic acid) in 50% (v/v) ACN and 0.1% (v/v) TFA. The spot proteins were identified from the peptide mass fingerprints obtained following MALDI‐TOF/TOF using MASCOT with MS/MS spectra from selected peptides. MS/MS searches were conducted against the nrNCBI database with search parameters: enzyme was trypsin; allowance of one missed cleavage site; fixed modification was carbamidomethyl (cysteine); variable modification was oxidation of Met; monoisotopic mass values; protein mass unrestricted; ±200 ppm as peptide mass tolerance; and ±1 Da as fragment mass tolerance.

## Results

### Muscle proteome 2‐DE analysis

**Figure 1:**
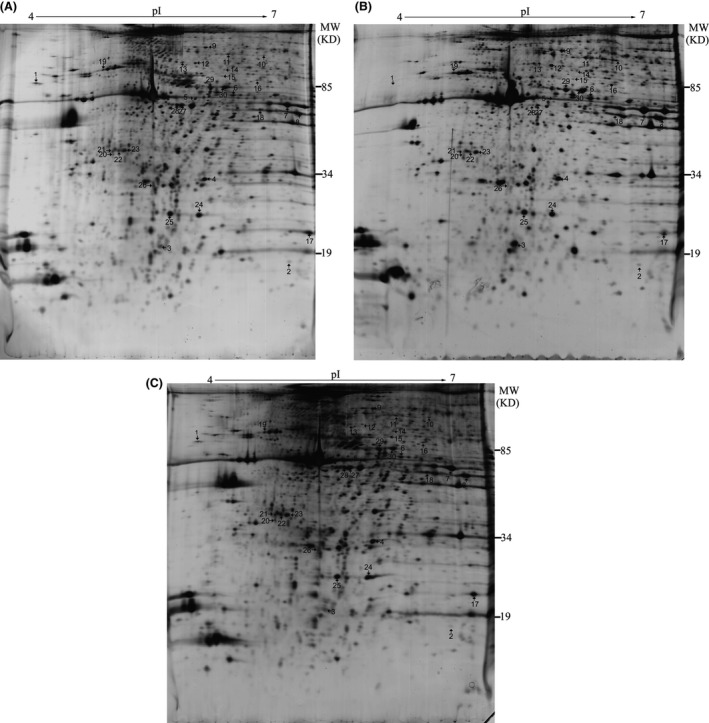
Separation of the abalone muscle proteins using 2‐DE. Representative 2‐DE images from (A) JJ muscle proteins, (B) TT muscle proteins, and (C) VV muscle proteins.

In this study, most protein spots were present on all 2‐DE gels or in all biological replicates of at least two of the abalone samples (representative 2‐DE gel Fig. 1). There were 922 ± 21 stained spots (three biological replicate gels, *n* = 3) for the JJ, 904 ± 25.6 stained spots (*n* = 3) for the TT and 936 ± 16.2 stained spots (*n* = 3) for the VV, with 254 differentially expressed spots identified in PDQuest 8.0. Additionally, the percentage volume of 85 spots varied more than twofold, with seven unique spots identified in JJ, three identified in VV, and two identified in TT.

### Analysis of “spots” and “genotypes”

Intensity variations between spots in the same locations can be attributed to a phenotypic variation, thus the use of protein spots detected in all gels helps to avoid any variation artifacts (Diz and Skibinski 2007). To compare protein expression among the three populations, 508 spots were chosen from gels, with selected spots being similar to previous experiments (Mosquera et al. 2003). A fully factorial ANOVA (type III sums of squares) was carried out according to previous recommendations using “spot” and “genotype” factors (Jin et al. 2001; Karp et al. 2005), with the “genotype” being a generic term for the three populations (JJ, TT and VV). ANOVA results are shown in Table 1 and both the “genotype” and “spot” were significant (*P* < 0.05). There was a striking difference among the spot intensity of three geographical populations (*P* < 0.01). Within each genotype, there is significant heterogeneity in variance values between spots, and the observation that the variance of spot volume differs between spots can be explained in several ways. Variation between protein genes might be involved, while variation between proteins in environmental plasticity might also be involved. There was a significant difference among the genotypes of three geographical populations (*P* < 0.05), indicating that the expression pattern is significantly different from the mean of the three species.

**Table 1:** ANOVA analyses

Source	Type III sum of squares	df	Mean square	*F*	Sig.
Corrected model	28,415.637	509	55.826	6.206	0.000
Intercept	16,916.032	1	16,916.032	1880.632	0.000
Spots	28,336.351	507	55.890	6.214	0.000
Genotype	79.285	2	39.643	4.407	0.012
Error	9120.793	1014	8.995		
Total	54,452.462	1524			
Corrected total	37,536.430	1523			

*R* ^2^ = 0.757 (Adjusted *R* ^2^ = 0.635).

### Genetic distances of the three geographical populations

**Figure 2:**
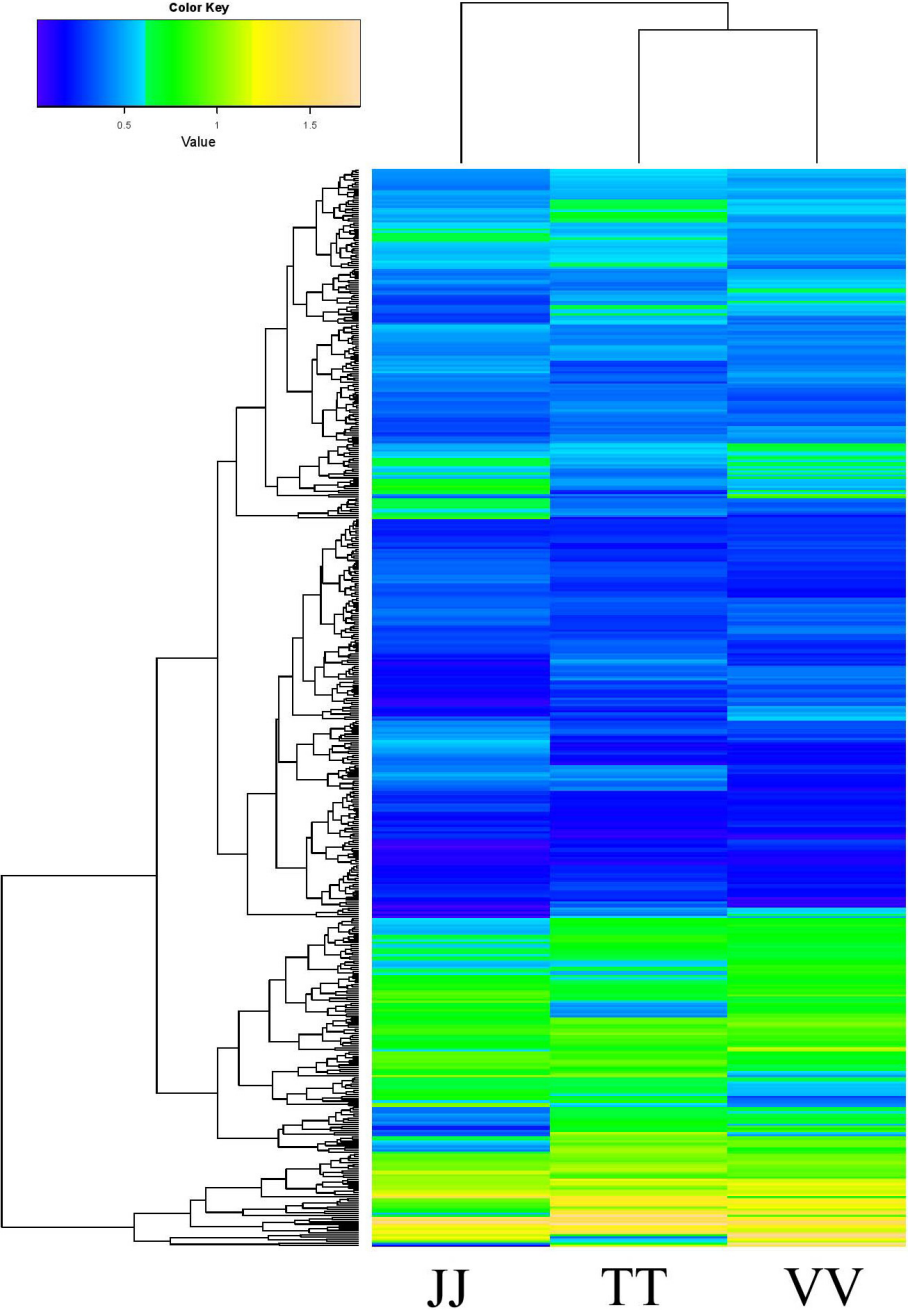
Dendrogram of hierarchical cluster analysis of JJ, TT, and VV.

Spot variations that were always present in all the individuals in the comparison were examined. Relations between “genotypes” were examined via hierarchical clustering, with 508 normalized spots examined. TT and VV clustered together and then were clustered with JJ, with the distance between JJ and VV being the maxima according to hierarchical cluster analysis (Fig. 2).

### Identification of differentially expressed protein spots via 2‐DE

A total of 30 protein spots were identified using 2‐DE gels. Generally, a protein was identified based on at least two qualified MS/MS spectra, with protein identifications summarized in Table S1. Differentially expressed proteins were determined using normalized % spot volumes (Table 2) and matched to specific functions or processes using the Gene Ontology (GO) database (www.geneontology.org) and http://www.uniprot.org/uniprot/. Most of the differentially expressed proteins were involved in contraction and protein regulation of muscle, response to stress and energy production and storage. The proteins involved in muscle contraction and regulation included actin, calponin, muscle myosin heavy chain, paramyosin, troponin T, and troponin I. Spot expression was higher in VV, which included almost every group for muscle contraction and regulation, while most of the protein expression in TT was lowest.

**Table 2:** Protein spots with important physiological functions identified by MS/MS in abalone muscle using normalized % spot volumes based on protein expression

No^a^	Proteins name^b^	Type	(Spot volume)% (Mean ± SD)			Physiological functions
			JJ	VV	TT	
Contraction and regulation proteins of muscle						
20	Actin	JJ > VV > TT	1.11 ± 0.03	0.20 ± 0.01	–	ATP binding protein binding
22	Actin 88F	VV > JJ > TT	4.70 ± 0.21	5.56 ± 0.13	–	ATP binding protein binding
1	Actin depolymerization factor/cofilin	VV > JJ > TT	1.11 ± 0.20	1.20 ± 0.03	–	Actin binding
17	Actin depolymerization factor/cofilin	JJ > VV > TT	21.79 ± 3.04	19.84 ± 2.2	2.44 ± 0.31	Actin binding
3	Actin‐2	TT > VV > JJ	5.25 ± 0.36	20.61 ± 2.68	27.09 ± 4.21	ATP binding protein binding
10	Calponin	TT > VV > JJ	0.70 ± 0.01	0.92 ± 0.24	1.45 ± 0.27	
11	Muscle myosin heavy chain	JJ > VV > TT	2.65 ± 0.25	1.65 ± 0.05	1.33 ± 0.01	Motor protein,Muscle protein,Myosin
14	Muscle myosin heavy chain	TT > VV > JJ	0.53 ± 0.04	0.62 ± 0.03	1.15 ± 0.13	Motor protein, Muscle protein, Myosin
5	Paramyosin	VV > JJ > TT	2.99 ± 0.17	3.68 ± 0.31	1.42 ± 0.34	Motor protein, Muscle protein, Myosin
29	Paramyosin	JJ > VV > TT	14.61 ± 0.69	8.68 ± 0.25	6.05 ± 0.51	Motor protein, Muscle protein, Myosin
16	PREDICTED:similar to fast myosin heavy chain HCII	VV > JJ > TT	1.11 ± 0.06	1.22 ± 0.04	–	Motor protein, Muscle protein
23	RecName:Full = Actin	VV > JJ > TT	6.82 ± 0.19	7.87 ± 0.53	3.50 ± 0.17	ATP binding protein binding
25	RecName:Full = Troponin I;Short = TnI	VV > JJ > TT	18.60 ± 1.13	21.05 ± 3.39	8.32 ± 0.54	Muscle proteinactin binding/muscle cell homeostasis
15	Troponin I	TT > JJ > VV	0.68 ± 0.07	–	0.73 ± 0.09	
24	Troponin I	VV > JJ > TT	17.75 ± 1.23	17.83 ± 2.14	8.57 ± 0.72	Muscle proteinactin binding/muscle cell homeostasis
27	Troponin T	VV > JJ > TT	11.69 ± 1.37	13.33 ± 1.26	4.59 ± 0.03	Muscle protein/cellular calcium ion homeostasis, mesoderm development, mitochondrion organization, muscle cell homeostasis, muscle organ morphogenesis, sarcomere organization
28	Troponin T	VV > JJ > TT	8.22 ± 0.56	8.98 ± 0.42	1.76 ± 0.26	
Proteins involved in stress response						
9	Heat shock protein Hsp70	JJ > VV > TT	3.58 ± 0.08	3.47 ± 0.03	2.04 ± 0.29	ATP binding/Stress response
2	Cu/Zn‐superoxide dismutase	VV > JJ > TT	1.06 ± 0.01	1.04 ± 0.04	0.36 ± 0.02	Metal ion binding/oxidation reduction, superoxide metabolic process
Proteins involved in energy metabolism						
19	ATP synthase beta subunit	TT > VV > JJ	1.94 ± 0.05	3.12 ± 0.13	3.46 ± 0.28	ATP binding, Hydrolase/Ion transport
8	Fructose 1, 6‐bisphosphate aldolase	VV > TT > JJ	12.66 ± 1.52	28.05 ± 3.19	15.94 ± 2.56	
18	Fructose 1, 6‐bisphosphate aldolase	VV > JJ > TT	1.11 ± 0.02	1.22 ± 0.19	–	
12	Hypothetical protein CC1G_00866	VV > JJ > TT	2.58 ± 0.03	3.29 ± 0.14	1.30 ± 0.24	Magnesium ion binding, intramolecular transferase activity, phosphotransferases/carbohydrate metabolic process
13	Hypothetical protein MGL_1163	VV > JJ > TT	2.32 ± 0.14	2.43 ± 0.37	–	Intramolecular transferase activity, phosphotransferases, magnesium ion binding/carbohydrate metabolic process
26	RecName: Full = arginine kinase; Short = AK	VV > JJ > TT	2.36 ± 0.12	3.46 ± 0.34	–	ATP binding, arginine kinase activity
7	RecName:Full = Arginine kinase;Short = AK	VV > JJ > TT	6.99 ± 0.61	53.42 ± 6.58	4.61 ± 0.39	ATP binding, arginine kinase activity
30	RecName:Full = Enolase;AltName:Full = 2‐phosphoglycerate dehydratase;AltName:Full = 2‐phospho‐D‐glycerate hydro‐lyase	JJ > VV > TT	19.15 ± 2.43	17.11 ± 2.01	6.48 ± 0.46	Lyase/Glycolysis
6	Tauropine dehydrogenase	JJ > VV > TT	5.44 ± 0.35	3.24 ± 0.05	3.23 ± 0.37	NAD or NADH binding, oxidoreductase activity, acting on the CH‐OH group of donors, NAD or NADP as acceptor/glycerol‐3‐phosphate catabolic process, oxidation reduction
4	Triosephosphate isomerase	JJ > VV > TT	13.12 ± 1.43	12.59 ± 1.63	9.99 ± 0.61	Isomerase/Fatty acid biosynthesis, Gluconeogenesis, Glycolysis, Lipid synthesis, Pentose shunt
Other proteins						
21	PREDICTED:hypothetical protein XP_533132	JJ > VV > TT	1.66 ± 0.02	1.49 ± 0.13	–	

a Spot number corresponds to the number on the 2‐DE in Figure 1.

b Proteins identified by de novo sequencing and MASCOT, a NCBI nonredundant database.

Some protein spots were involved in energy production and storage, including ATP synthase *β* subunit, fructose bisphosphate aldolase, arginine kinase, enolase, triosephosphate isomerase, and tauropine dehydrogenase. The expression patterns of interest included, spot 19 (ATP synthase *β* subunit) which was TT > VV > JJ and spot 8 (fructose‐1,6‐bisphosphate aldolase) which was VV > TT > JJ. Protein spots with the expression pattern VV > JJ > TT included, spots 7 (arginine kinase), 12 (hypothetical protein), 13 (hypothetical protein), 18 (fructose‐1, 6‐bisphosphate aldolase), and 26 (arginine kinase); while spots with the expression pattern JJ > VV > TT included, spots 4 (triosephosphate isomerase), 6 (tauropine dehydrogenase), and 30 (enolase). Spots 8, 18, 12, 13, 26, and 7 had the highest expression in VV, while spots 30, 6, and 4 had the highest expression in JJ. The protein expression in the TT population was low in general, with the exception of spot 19. Furthermore, two stress proteins were identified, heat shock protein 70 (Hsp70) (JJ > VV > TT) and Cu/Zn‐ superoxide dismutase (JJ > VV > TT).

### Subcellular location of identified proteins

The identified proteins were then matched to cellular component by searching GO (www.geneontology.org) and http://www.uniprot.org/uniprot/. With the bioinformatics analysis, subcellular locations of 30 identified gel spots were summarized. ATP synthase beta subunit was in the mitochondria, and other proteins that were mainly in the cytoplasm.

### Predicted interactions of identified differentially proteins from muscle

**Figure 3:**
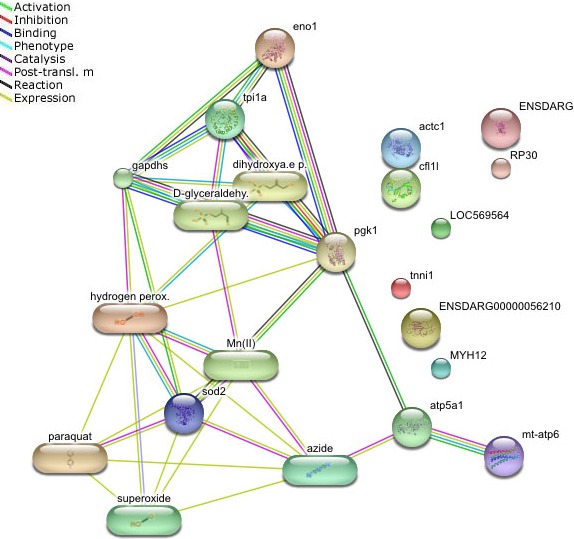
Predicted interactions of identified differentially proteins from muscle.

Predicted interactions of identified differentially proteins from muscle at http://stitch.embl.de/cgi/website were showed in Figure 3. Protein abbreviations and corresponding full name were showed in Table 3. Identified differentially proteins involved in the physiological pathway were showed in Table 4. Identified differentially proteins involved in major physiological pathway including Glycolysis/Gluconeogenesis, Inter‐pathway connection between “Glycolysis/Gluconeogenesis” and “Carbon fixation”, Oxidative phosphorylation.

**Table 3:**
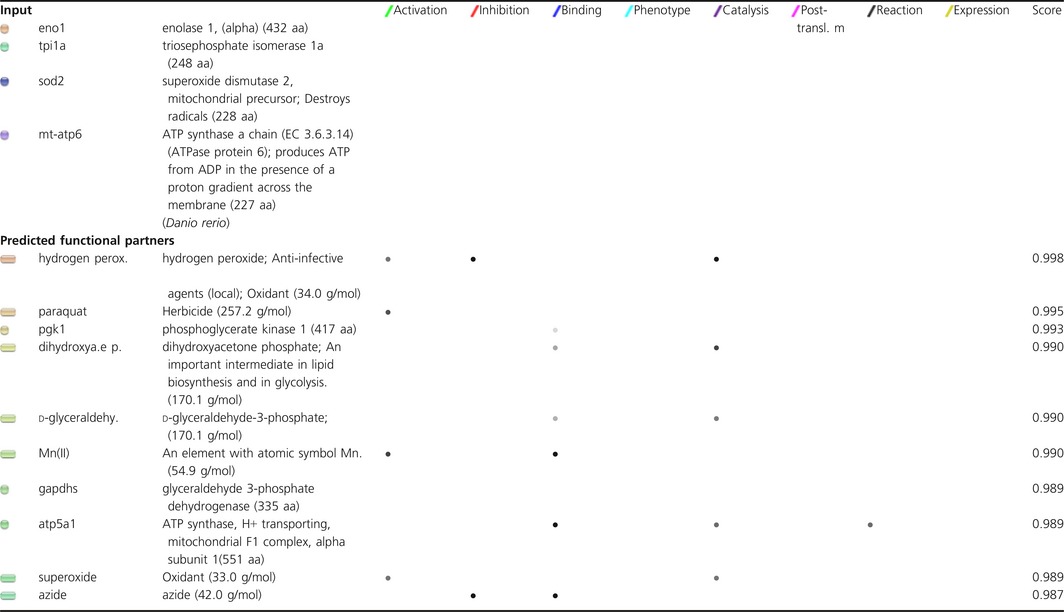
Protein abbreviations and corresponding full name

**Table 4:** Identified differentially proteins involved in the physiological pathway

Annotated pathway (KEGG) name	Items
Glycolysis/Gluconeogenesis	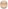 eno1 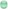 tpi1a 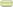 dihydroxyacetone phosphate 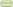 d‐glyceraldehyde‐3‐phosphate 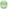 gapdhs
Inter‐pathway connection between “Glycolysis/Gluconeogenesis” and “Carbon fixation”	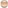 eno1 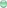 tpi1a
Oxidative phosphorylation	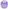 mt‐atp6 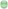 atp5a1

## Discussion

### Genetic distances of the three geographical populations

Protein expression profiles were determined in three geographical abalone populations using high‐resolution 2‐DE, with the results identifying both “genotype” and “spot” significances. Following hierarchical clustering, TT and VV were clustered together and then clustered with JJ based on genetic distances. The wild (Japanese) and cultured (Taiwanese and Vietnamese) populations of small abalone were examined using seven microsatellite loci to assess the degree of genetic differentiation among them (our team research papers You 2009; You et al.^2011^). The result showed that the largest distance was between the Japanese and Vietnamese populations (You 2009; You et al.^2011^). The present result was consistent with their geographical location. Moreover, these clustering results were consistent with estimated genetic variations between populations as determined by microsatellite DNA markers (You 2009). Thus, proteomics may be used as a different strategy to resolve taxonomic problems and aid in determining biogeographical distributions of these species.

### Contraction and protein regulation in muscle

The differences in protein expression pertaining to muscle contraction and muscle protein regulation are difficult to interpret. Spot intensities were higher in VV, to include almost every group of proteins pertaining to muscle contraction and muscle protein regulation, while spot intensities in TT were low. These differences could be attributed to possible differences in the properties of the abalone foot or related to the foot glandular and accessory organs (Chen et al. 1995; Greenfield et al. 1998). Thus, myosin heavy chain is required for the function of myosin (Patwary et al. 1999). The expression level of actin can be related to the rearrangement of both intrachain disulfide bonds (López et al. 2002).

### Proteins associated with energy production and storage

We identified several types of proteins associated with energy production and storage including the ATP synthase *β* subunit, fructose‐1,6‐bisphosphate aldolase, arginine kinase, triosephosphate isomerase, enolase, and tauropine dehydrogenase. Spot 19 was identified as the ATP synthase *β* subunit, a key enzyme in cellular energy interconversion. Protein spots 8 and 18 were identified as fructose‐1, 6‐bisphosphate aldolase, a key enzyme in glycolysis and gluconeogenesis (Rutter 1964). Spots 7 and 26 were identified as arginine kinase, which plays a crucial role in energy metabolism in invertebrates. Lastly, spot 30 was identified as enolase, which is one of key enzyme in glycolysis and its main activity is in the metabolism of carbohydrates (Duncan et al. 2012).

In this study, spots 8, 18, 12, 13, 26, and 7 showed the highest intensities in the VV population, while spots 30, 6, and 4 showed the highest intensities in the JJ population. The expression pattern of spot 19 (ATP synthase *β* subunit) was TT > VV > JJ, with otherwise low intensities noted for the TT population. Common features are low activities of aerobic ATP production, relatively high activities of arginine kinase, tauropine, and d‐lactate dehydrogenases (Baldwin et al. 2007). Previously, shell lengths of the Japanese and Taiwanese populations were shown to be 7.48% and 15.72% larger than that of Vietnamese population at Day 420 (You et al. 2009). It is difficult to explain the differences between the three populations regarding the expression of energy production and storage proteins relating to shell lengths. For the entire rearing period, these differences may be related to energy allocation, with a smaller fraction of energy allocated to growth in Vietnamese population. Based on this preliminary data and the limited protein information, it may be difficult to establish an association between abalone muscle functions and shell length.

### Proteins associated with stress response

Spots 9 was identified as Hsp70, a highly conserved stress protein that can protect cells from harmful assaults. Hsp70 is a member of molecular chaperones. Hsp70 expression levels show stress tolerance, with Hsp70 aiding in the correct folding of nascent polypeptides and targeting damaged proteins for proteolytic destruction (López et al. 2002). Hsp70 also plays an important role in antiapoptotic effects and antitumor immune therapy. Hsp70 was significantly differentially expressed between the three abalone populations. The fact that Hsp70 had an expression pattern of JJ > VV > TT, these results may provide evidence that three abalone populations were physiologically differentiated to temperature and could be similar to a study where two mollusk (*M. galloprovincialis* and *M*. *edulis*) species were physiologically differentiated to temperature (Hilbish et al. 1994).

Spot 2 was identified as Cu/Zn‐superoxide dismutase (Cu/Zn‐SOD), which is closely related to immunity in mollusks. It can increase phagocytic cell activity and immune function and protect the cell from ROS poisoning (Kim et al. 2007). The expression pattern of Cu/Zn‐SOD was JJ (1.057) > VV (1.036) > TT (0.36). A study by Wang (2010) showed that the SOD activity of hemolymph in the Japanese population was significantly higher than that of the Taiwanese population, which is consistent with this study.

According to You et al. (2009) study, for the entire rearing period, the survival rate was 78.3 ± 5.34% for JJ, 12.6 ± 4.13% for TT, and 15.7 ± 4.62% for VV. Therefore, Hsp70 and Cu/Zn‐SOD expression levels may be related to disease resistance and play a role in population survival rates.

## Conclusion

This study examined 2‐DE generated protein expression profiles in three different geographical populations of *H. diversicolor*. Examining the “genotype” showed that all of the three populations were significant (*P* < 0.05). Hierarchical clustering showed that TT and VV clustered together, followed by clustering with JJ, which is consistent with their geographical locations. This study showed that proteomic approaches are useful in identifying unknown and differentially expressed protein associated with physiological processes within different populations.

## Conflict of Interest

None declared.

## Supporting information


**Table S1.** Protein identification using MASCOT database searches.
