# Supplementary material for: Protein changes in abalone foot muscle from three geographical populations of Haliotis diversicolor based on proteomic approach
Source: Ecol Evol. 2016 Apr 27;6(11):3645–57. doi: 10.1002/ece3.2128 (PMC4851958; doi:10.1002/ece3.2128)
Supplement: Supplementary file 1 — Table S1. Protein identification using MASCOT database searches. [file ECE3-6-3645-s001.doc]

Supplementary Table 1 Protein identification using MASCOT database searches:

| **Spot no^a)^** | **Protein ^b)^** | **Species ^c)^** | **Accession**  **No ^c)^** | **Protein**  **MW** | **Protein**  **PI** | **Protein**  **Score** | **Protein**  **Score C.I%** | **Rank Result Type** |
| --- | --- | --- | --- | --- | --- | --- | --- | --- |
| 1 | actin depolymerisation factor/cofilin | *Haliotis diversicolor* | gi|15707278 | 18306.2 | 6.74 | 390 | 100 | Mascot |
| 2 | Cu/Zn-superoxide dismutase | *Haliotis diversicolor* | gi|166406955 | 12622.2 | 5.34 | 94 | 99.547 | Mascot |
| 3 | actin-2 | *Lotharella amoeboformis* | gi|15216717 | 27210.8 | 5.25 | 93 | 99.48 | Mascot |
| 4 | triosephosphate isomerase | *Tenebrio molitor* | gi|22090453 | 27004.9 | 6 | 138 | 100 | Mascot |
| 5 | paramyosin | *Schistosoma mansoni* | gi|161055 | 51288.7 | 5.21 | 86 | 97.331 | Mascot |
| 6 | tauropine dehydrogenase | *Haliotis discus hannai* | gi|20804397 | 45435.2 | 5.34 | 128 | 100 | Mascot |
| 7 | RecName:Full=Arginine kinase；Short=AK | *Haliotis madaka* | gi|1708614 | 40245.1 | 5.73 | 228 | 100 | Mascot |
| 8 | fructose 1，6-bisphosphate aldolase | *Haliotis diversicolor* | gi|166406769 | 24196.2 | 7.08 | 559 | 100 | Mascot |
| 9 | heat shock protein Hsp70 | *Antheraea yamamai* | gi|47232576 | 69834.8 | 5.7 | 388 | 100 | Mascot |
| 10 | Calponin | *Haliotis diversicolor* | gi|156144968 | 24185.2 | 9.18 | 228 | 100 | Mascot |
| 11 | muscle myosin heavy chain | *Sepia esculenta* | gi|189007784 | 223220 | 5.49 | 91 | 99.116 | Mascot |
| 12 | hypothetical protein CC1G_00866 | *Coprinopsis cinerea okayama7#130* | gi|169849231 | 61388.5 | 5.56 | 179 | 100 | Mascot |
| 13 | hypothetical protein MGL_1163 | *Malassezia globosa CBS 7966* | gi|164661545 | 59815.1 | 5.81 | 184 | 100 | Mascot |
| 14 | muscle myosin heavy chain | *Sepia esculenta* | gi|189007784 | 223220 | 5.49 | 175 | 100 | Mascot |
| 15 | troponin I | *Mizuhopecten yessoensis* | gi|2541916 | 34706.5 | 5.69 | 109 | 99.986 | Mascot |
| 16 | PREDICTED:similar to fast myosin heavy chain HCII | *Taeniopygia guttata* | g i|224074785 | 224217.1 | 5.68 | 117 | 99.998 | Mascot |
| 17 | actin depolymerisation factor/cofilin | *Haliotisd Iversicolor* | gi|157072781 | 18306.2 | 6.74 | 809 | 100 | Mascot |
| 18 | fructose 1，6-bisphosphate aldolase | *Haliotisd iversicolor* | gi|166406769 | 24196.2 | 7.08 | 450 | 100 | Mascot |
| 19 | ATP synthase beta subunit | *Haliotis rufenscens* | gi|71370914 | 46238 | 4.9 | 1020 | 100 | Mascot |
| 20 | actin | *Vannella ebro* | gi|33946345 | 42031.9 | 5.23 | 488 | 100 | Mascot |
| 21 | PREDICTED:hypothetical protein XP_533132 | *Canis lupus familiaris* | gi|73964667 | 42052.9 | 5.24 | 524 | 100 | Mascot |
| 22 | actin 88F | *Drosophila mauritiana* | gi|294714383 | 32933.3 | 5.24 | 661 | 100 | Mascot |
| 23 | RecName:Full=Actin | *Thermomyces lanuginosus* | gi|113307 | 41837 | 5.44 | 400 | 100 | Mascot |
| 24 | troponin I | *Chlamys nipponensis akazara* | gi|2668408 | 32106.2 | 5.48 | 113 | 99.995 | Mascot |
| 25 | RecName:Full=Troponin I；Short=TnI | *Chlamys nipponensis akazara* | gi|47117348 | 34672.5 | 5.69 | 107 | 99.978 | Mascot |
| 26 | RecName: Full=arginine kinase； Short=AK | *Haliotis madaka* | gi|1708614 | 39788.9 | 5.73 | 167 | 100 | Mascot |
| 27 | troponin T | *Haliotis diversicolor* | gi|166406876 | 18030.2 | 5.68 | 162 | 100 | Mascot |
| 28 | troponin T | *Haliotis diversicolor* | gi|166406876 | 18030.2 | 5.68 | 99 | 99.733 | Mascot |
| 29 | paramyosin | *Schistosoma mansoni* | gi|161055 | 51288.7 | 5.21 | 89 | 98.723 | Mascot |
| 30 | RecName:Full=Enolase；AltName:Full=2-phosphoglycerate dehydratase；AltName:Full=2-phospho-D-glycerate hydro-lyase | *Loligo peale* | g i|3023702 | 47738.3 | 5.78 | 363 | 100 | Mascot |

a) Spot number corresponds to the number on the 2DE in Fig. 1.

b) Protein identiﬁed by the de novo sequencing and MASCOT (www.matrixscience.com) from the NCBI nonredundant database.

c) Species and accession number from the NCBI nonredundant.
